# Approach to the Patient: Diagnostic Challenges in the Workup for Polycystic Ovary Syndrome

**DOI:** 10.1210/clinem/dgae910

**Published:** 2025-01-21

**Authors:** Anju E Joham, Chau Thien Tay, Joop Laven, Yvonne V Louwers, Ricardo Azziz

**Affiliations:** Monash Centre for Health Research and Implementation, Faculty of Medicine Nursing and Health Sciences, Monash University, Clayton 3168, Australia; Departments of Diabetes and Endocrinology, Monash Health, Clayton 3168, Australia; Monash Centre for Health Research and Implementation, Faculty of Medicine Nursing and Health Sciences, Monash University, Clayton 3168, Australia; Departments of Diabetes and Endocrinology, Monash Health, Clayton 3168, Australia; Division of Reproductive Endocrinology and Infertility, Department of Obstetrics and Gynaecology, Erasmus Medical Centre, Rotterdam 3015 GD, The Netherlands; Division of Reproductive Endocrinology and Infertility, Department of Obstetrics and Gynaecology, Erasmus Medical Centre, Rotterdam 3015 GD, The Netherlands; Departments of Obstetrics and Gynecology, and Medicine, University of Alabama at Birmingham, Birmingham, AL 35243, USA

**Keywords:** polycystic ovary syndrome, PCOS, diagnosis

## Abstract

Polycystic ovary syndrome (PCOS) affects 10% to 13% of women globally. It is a condition with metabolic, reproductive, and psychological features, with health impacts across the lifespan. The etiology of PCOS is complex, with an interplay of several factors, including genetic and epigenetic susceptibility, androgen exposure in early life and adiposity-related dysfunction leading to hypothalamic-ovarian disturbance. Diagnosis is recommended based on the International PCOS Guideline criteria, with diagnosis confirmed in adults when 2 of out the following 3 criteria are met: (i) hyperandrogenism (clinical or biochemical); (ii) irregular cycles; and (iii) polycystic ovary morphology or elevated anti-Müllerian hormone (AMH) levels. With its clinical heterogeneity, distinct phenotypes, variation across the lifespan and ethnic variation, PCOS diagnosis can present significant diagnostic challenges to clinicians.

Polycystic ovary syndrome (PCOS) is a complex heterogeneous multisystem disorder affecting 10% to 13% of reproductive-aged women (1) and 3% to 11% of adolescent girls (2), depending on the population studied and the diagnostic criteria applied. The cardinal hormonal abnormalities in PCOS are hyperandrogenism and insulin resistance. PCOS has significant metabolic (obesity, metabolic syndrome, impaired glucose tolerance, type 2 diabetes (3), cardiovascular risk factors), reproductive (anovulation, menstrual dysfunction, subfertility) (4), dermatological (hirsutism, acne, female pattern hair loss) (5), and psychological (depression, anxiety (6), eating disorders (7), impaired quality of life) (8, 9) sequelae. The 2023 International PCOS Guideline criteria revises the Rotterdam criteria and now recommends diagnosis in adults to be based on the presence of 2 of 3 features: oligo/amenorrhea, clinical/biochemical hyperandrogenism, and polycystic ovary morphology (PCOM) on ultrasound or elevated anti-Müllerian hormone (AMH). For adolescents, a diagnosis requires both oligo/amenorrhea and clinical/biochemical hyperandrogenism.

PCOS diagnosis requires the exclusion of mimicking disorders that can result in ovulatory dysfunction and/or hyperandrogenism (10, 11). Clinical evaluation should consider Cushing syndrome, androgen-secreting neoplasms, syndromes of severe insulin resistance and lipodystrophy, and acromegaly. Laboratory investigations should include the measurement of thyroid stimulating hormone (TSH) to exclude thyroid disorders, prolactin to exclude hyperprolactinemia, and 17-hydroxyprogesterone (17-OHP), obtained basally and in the pre-ovulatory or follicular phase of the menstrual cycle to exclude 21-hydroxylase deficient non-classic congenital adrenal hyperplasia (NCAH) (11), given that NCAH is unable to be distinguished clinically from PCOS (12). A diagnostic algorithm is presented in Fig. 1.

**Figure 1.**
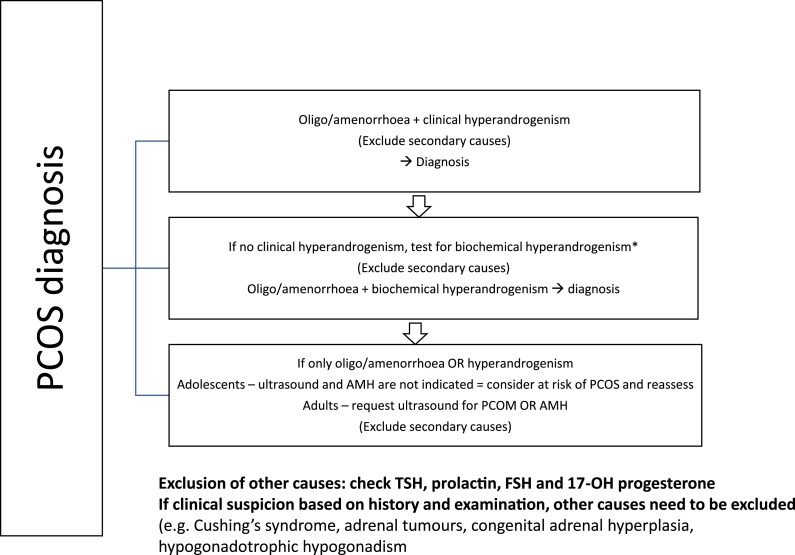
PCOS diagnostic algorithm. Abbreviations: AMH, anti-Müllerian hormone; FSH, follicle stimulating hormone; PCOM, polycystic ovary morphology; TSH, thyroid stimulating hormone. Adapted from the 2023 International Evidence-Based Guideline on Polycystic Ovary Syndrome.

PCOS diagnosis is complex with the diagnostic criteria generating 4 clinical phenotypes in adults: phenotype A (featuring hyperandrogenism, ovulatory dysfunction, and PCOM), phenotype B (hyperandrogenism and ovulatory dysfunction without PCOM), phenotype C (hyperandrogenism and PCOM without ovulatory dysfunction), and phenotype D (ovulatory dysfunction and PCOM without hyperandrogenism) (13, 14). In addition, clinical features of PCOS are heterogeneous with manifestations varying across individuals and the reproductive life stage. While clinical presentation in PCOS does not necessarily correlate with genotype, subdividing PCOS patients by key clinical presentation, such as the presence or absence of hyperandrogenism, ovulatory dysfunction and PCOM (ie, phenotypes A-D) is helpful to determine what diagnostic testing may be required. For example, women with clinical evidence of hyperandrogenism (eg, hirsutism) and ovulatory dysfunction require only the exclusion of mimicking disorders for the diagnosis of PCOS. Alternatively, women who present solely with menstrual dysfunction may require assessment of ovarian morphology and/or circulating androgens, and if either of these is abnormal, then exclusion of mimicking disorders. Meanwhile, women presenting with what appears to be solely hirsutism merit being evaluated by ovarian ultrasonography or AMH levels, before assessing for mimicking disorders.

## Ovulatory Dysfunction

Clinically, ovulatory dysfunction is marked by significant and chronic menstrual dysfunction, including polymenorrhea (ie, episodes of menstrual-like vaginal bleeding occurring at < 21-day intervals) or oligo-amenorrhea (ie, episodes of vaginal bleeding occurring at > 35-day intervals or < 8 times yearly). However, apparent regularity of menstrual cycles (ie, self-reported eumenorrhea) in the presence of hyperandrogenism is associated with ovulatory dysfunction in up to 40% of presenting patients (15). Women with PCOS often experience longer menstrual cycle intervals because follicle development is partially arrested by lower sensitivity to follicle stimulating hormone (FSH), contributed to in part by elevated levels of AMH (16). Moreover, increased gonadotropin hormone-releasing hormone (GnRH) pulse amplitude and frequency results in the pituitary secreting high levels of luteinizing hormone (LH) and relatively low levels of FSH (17). High LH serum levels further contribute to increased androgen production in the ovary, thereby causing follicular arrest. This results in ovulatory dysfunction, which is characterized by infrequent or prolonged menstrual cycles. Women with a more severe phenotype may experience amenorrhea. The average cycle length for women with anovulation is 60 days, while it is 43 days for women with oligo-ovulation (18).

Defining oligomenorrhoea in adolescents is complex, as irregular cycles and ovulatory dysfunction are a normal component of pubertal transition, with physiological maturation of the hypothalamic, pituitary ovarian axis occurring over several years. The International PCOS Guideline recommends that in adolescents, irregular menstrual cycles are defined according to gynecological age (11):

- Irregular cycles are normal in the first year post menarche as part of pubertal transition
- > 1 to < 3 years post menarche: < 21 or > 45 days
- > 3 years post menarche: < 21 or > 35 days or < 8 cycles per year
- > 1 year post menarche > 90 days for any one cycle
- Primary amenorrhea by age 15 or > 3 years post thelarche

## Hyperandrogenism

Another cardinal feature of PCOS is androgen excess, also referred to as hyperandrogenism, which is present in the majority of patients with the disorder. In a meta-analysis of 41 original studies enrolling a total of 13 796 patients with PCOS we observed that < 20% do not exhibit hyperandrogenism, whether detected in the clinical setting or in medically unbiased populations (19). Notably, in addition to being part of the diagnostic algorithm (11), the degree of hyperandrogenism is associated with the risk of metabolic dysfunction and associated cardiometabolic morbidities of PCOS (20-22). The presence of hyperandrogenism can be determined either clinically or biochemically.

### Clinical Hyperandrogenism

The most common clinical sign of hyperandrogenism is the presence of hirsutism (ie, excess facial or body terminal hair growth in a male-like pattern in a female). Most women with hirsutism have demonstratable biochemical hyperandrogenemia (23). Alternatively, and as indicated by the 2023 International PCOS Guideline (24), female pattern hair loss (FPHL) or acne, if in isolation (ie, without hirsutism), are relatively weak indicators of hyperandrogenemia (5).

**Figure 2.**
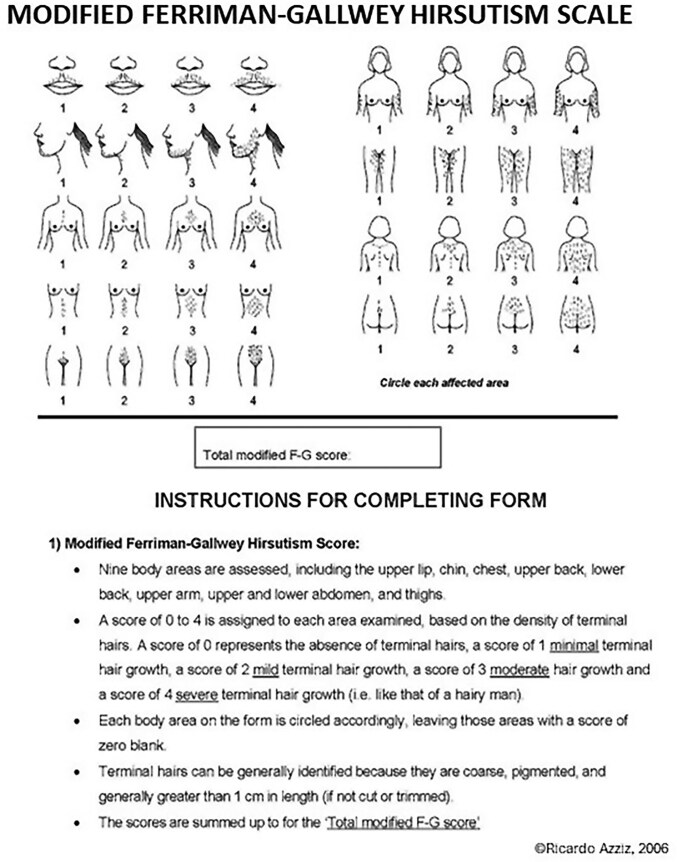
Modified Ferriman-Gallwey Hirsutism scale. Reproduced with permission.

While there are a number of methods to assess patients for hirsutism, the most commonly used, and the one recommended by the 2023 International PCOS Guideline (11), is the modified Ferriman-Gallwey (mFG) visual scale (25). Originally described by Ferriman and Gallwey (26), the mFG scale assesses terminal hair growth in 9 body areas, with each area assigned a score of 0 (no terminal hairs observed) to 4 (a density of terminal hairs consistent with that of a mature male), and the individual scores summed to provide the total mFG score (Fig. 2). Importantly, when examining patients for hirsutism, clinicians should strive to only assess the presence of terminal, not vellus hairs. While the use of the scale is somewhat subjective, reference to a published visual atlas may be helpful in standardizing measurements (25).

Where a full assessment is unable to be performed to calculate the mFG score, due to cultural limitations or patient preference, simplified approaches to assess the mFG score may be considered. Although the simplified versions of the mFG score may be useful, they do not have the same level of predictive value as the full mFG score (27-32). Moreover, all simplified scores developed include either examination of the abdomen or the thighs or both, which may still not be acceptable in these patient groups. Another option is for a self-completed mFG score, where the patient scores themselves. Most studies have noted that while a self-reported mFG score correlates somewhat with the clinician-determined mFG score, it had low specificity, limiting its application (27), or had significant discordance (33-35). It should be noted that these studies assessed generally smaller cohorts of women with PCOS or those seen in the clinical setting. Consequently, while a self-assessed excess mFG score may be useful to predict hirsutism and/or PCOS, it is less accurate when compared to scoring determined by a trained clinician. Another option to consider is self-reported hirsutism, where women are asked whether they experience excess unwanted male-like hair growth is highly predictive of hirsutism and/or PCOS (36-38).

How much terminal hair growth in the 9 body areas assessed by the mFG scale is too much, reflecting hirsutism, and how much is normal variation? The current 2023 International PCOS Guideline suggests that an mFG “of 4-6 should be used to detect hirsutism, depending on ethnicity, acknowledging that self-treatment is common and can limit clinical assessment.” While prior recommendations suggested that a total mFG score of 8 or greater was indicative of hirsutism (39), these suggestions stemmed from a misinterpretation of Ferriman and Gallwey's original data, in which this value equaled the 95th percentile of the (presumably mostly White) population assessed by the investigators (26). However, there is no biological basis for using a 95th percentile as diagnostic cutoff for hirsutism. Rather, diagnostic cutoff values should be determined using cluster analyses and other approaches to determining natural subpopulations in a cohort of medically unbiased individuals. In fact, Ferriman himself recognized this, preferring to define women as “hirsute when body hair scores exceeded 4 and non-hirsute when less (40).” We and others have used cluster analysis in medically unbiased populations to identify the mFG value that defines hirsutism (41-43), reporting that values between 3 and 5 were the minimal mFG cutoffs for identifying women as hirsute. A similar strategy is being used to define hirsutism in various ethnic populations around the globe by the ongoing PCOS Phenotype in Unselected Populations (PPUP) Study (44).

### Biochemical Hyperandrogenism

Hyperandrogenism can also be established through the measurement of circulating androgens, although determination of which androgens to measure, what assay methods, and what cutoff values to use requires careful consideration. We should note that the 2023 International Guideline indicate that “the assessment of biochemical hyperandrogenism is of greatest value in patients with minimal or no clinical signs of hyperandrogenism (ie, hirsutism)” (11). As such, measuring circulating androgens will be most useful in non-hirsute women who either: (i) report menstrual irregularity; or (ii) complain of unwanted male-like hair growth, regardless of their mFG score (37). A recent study demonstrated that by following a similar diagnostic algorithm, only 15% of women evaluated for PCOS required biochemical measurement of androgens (45).

If androgens are needed to complete the diagnostic puzzle of PCOS, clinicians should use the highest quality and most accurate assays available to them, noting that accessibility will vary globally. We should note that the measurement of total or free testosterone, using direct immunoassay, is highly insensitive and inaccurate and is not recommended. The preferred methods for measuring total testosterone include mass spectrometry or high-quality immunoassay after extraction and chromatography (46). In turn, the amount of free testosterone in a sample can be estimated directly from the total testosterone measurement either by equilibrium dialysis, competitive binding, or ammonium sulfate precipitation (47-49)

Others suggest that free testosterone can be estimated by calculations that reflect the understood binding kinetics of testosterone to albumin and sex hormone binding globulin (SHBG) in the circulation (50-52). In addition, the free androgen index (FAI = 100 × [total testosterone/SHBG]) is another widely used calculation of testosterone bioavailability (51). However, the accuracy of the FAI is severely hampered if SHBG concentrations are less than 30 nmol/L (52), often found in women with PCOS. Furthermore, clinicians should recognize that calculated free testosterone does not actually reflect physiology, as it is based on assumptions that are not necessarily accurate, namely, that in all tissues only unbound testosterone is biologically active while testosterone bound to albumin, SHBG, and other proteins in the circulation is biologically inert (53). Consequently, clinicians should interpret and use calculated free testosterone measures with caution.

The 2023 International Guideline notes that “healthcare professionals should use total and free testosterone to assess biochemical hyperandrogenism in the diagnosis of PCOS…” and “if testosterone or free testosterone is not elevated, healthcare professionals could consider measuring androstenedione [A4] and dehydroepiandrosterone sulfate (DHEAS)… (11),” noting their poorer specificity. Measuring DHEAS and A4 may increase the proportion of patients determined to be hyperandrogenemic by 10% to 15% (54). The measurement of 11-oxygenated androgens (ie, 11-oxo-androgens such as 11-ketotestosterone, 11- ketoandrostenedione, 11β-hydroxyandrostenedione, and 11β-hydroxytestosterone) has been suggested to be of diagnostic value, although research is still ongoing (55-57). Clinicians should note that the measurement of circulating androgens is most useful in women without clinical evidence of hyperandrogenism, eg, hirsutism.

## Polycystic Ovarian Morphology

PCOM assessed by ultrasonography is considered as 1 of the 3 cardinal features of PCOS. The presence of PCOM is most often detected using transvaginal ultrasonography and is characterized by the presence in at least one ovary of > 20 pre-ovulatory or antral follicles (ie, measuring 2-9 mm in diameter) or increased ovarian volume (> 10 mL). The importance of PCOM as a diagnostic criterion for PCOS was reaffirmed in the current 2023 International PCOS Guideline (24). PCOM is also encountered in in women without PCOS ranging from 5% up to 50% especially in women younger than 30 years and in selected populations (58). The occurrence of PCOM in ovulatory women not showing clinical or biochemical androgen excess could be without any consequences, even though some studies suggest that isolated PCOM may represent the milder end of the PCOS spectrum (58). As previously noted, ovulatory dysfunction is a normal aspect of the pubertal transition, and similarly, the presence of PCOM is commonly observed in adolescents. Consequently, PCOM should not be used as a diagnostic feature of PCOS at less than 8 years post menarche. More than half of women with PCOM only also exhibit features of steroidogenic dysfunction, which suggests a PCOS carrier state (59).

In a recent diagnostic meta-analysis of 20 studies, pooling nearly 3900 control subjects and a similar number of PCOS patients, follicle number per ovary (FNPO) was the most accurate diagnostic marker for PCOM in adult women (60). However, due to a high risk of bias in patient selection and the lack of pre-defined thresholds in most studies, a specific cutoff could not be defined. The same study also identified 4 adolescent studies, where PCOM might also be a physiological phenomenon, and suggests weak evidence to support ovarian volume as a marker for PCOM, but appropriate cutoffs could not be defined. Moreover, a recent study in an unselected population cohort indicated that PCOM cutoffs are also dictated by ethnicity as well as by differences in age (61). It is thus crucial to determine population-specific normative ranges for PCOM in order to standardize the differences between women with and without PCOS across regionally distinct populations. Hence, there is an urgent need for age- and ethnicity-specific cutoffs for PCOM to aid the diagnosis of PCOS (24).

Determining FNPO is highly operator- and equipment-dependent, which affects its accuracy and reproducibility. Moreover, advances in equipment increase sensitivity and in turn FNPO cutoffs. However, ultrasound involves expensive equipment and trained personnel, leading to increased costs and impacts on accessibility. The route of the ultrasound to assess FNPO, whether transvaginal route or the transabdominal route, has impacts on accuracy. Lastly, in some women transvaginal ultrasound is unacceptable or may be perceived as invasive. Altogether these factors collectively limit the use, accuracy, and availability of ultrasound for the diagnosis of PCOM (62).

The presence of PCOM can now be inferred from elevated circulating level of AMH, although the exact diagnostic levels will vary by population and laboratory. A pelvic ultrasound has the advantage of detecting other abnormalities, including possible endometrial hyperplasia, and other pelvic pathology. Of note, the detection of PCOM, either ultrasound or AMH, should not be used for the evaluation of adolescents less than 8 years gynecological age with suspected PCOS.

## Anti-Müllerian Hormone

AMH is a polypeptide of the transforming growth factor beta (TGFβ) family, mainly secreted by granulosa cells of the pre-antral and small antral ovarian follicles. AMH levels correlate well with the FNPO in normal subjects as well as in women with PCOS, who generally display elevated levels of AMH (63). Hence, a single serum measurement of AMH has been proposed as a substitute to overcome all the issues associated with the ultrasound assessment of PCOM (62). Indeed, a recent meta-analysis, which included 82 studies, assessed the specificity and sensitivity of a single serum AMH measurement to define PCOM and thereby aid the diagnosis of PCOS (64). The aforementioned meta-analysis assessing FNPO by two-dimensional ultrasound reported a sensitivity of 79.3% and specificity of 83.2% for detecting PCOM (60). Similarly, the use of AMH levels as an indicator for PCOM showed a pooled estimates of 0.79 sensitivity and 0.87 specificity respectively (64). In adult women, there was good specificity as well as good sensitivity retrieved out of 68 individual studies with a moderate to low risk of bias. In adolescents, sensitivity and specificity data were less and also retrieved from a limited number of studies with considerable risk of bias. Hence, the authors of this paper support the use of AMH for defining PCOM in adults, in accordance with the diagnostic algorithm suggested by the 2023 International PCOS Guideline (13). However, AMH alone is insufficient for PCOS diagnosis and is nonspecific for PCOM in adolescents (64).

The diagnostic accuracy of ultrasound in assessing PCOM varies depending on confounders such as age, ethnic background, body mass index (BMI), and criteria and protocols used to diagnose PCOS (60). Similarly, AMH, since it correlates very well with the FNPO, will suffer from a similar diagnostic inaccuracy. Indeed, the recent international guideline also indicated that AMH levels are influenced by age, BMI, ethnicity and the assay used (11). Moreover, there still is no international reference making comparisons between different assays impossible. Definition of specific cutoffs for FNPO as well as for AMH is still problematic and there is an urgent need for age specific cutoffs for both parameters (65, 66).

## Diagnosis in Adolescents

Diagnosis of PCOS in adolescents is often challenging due to the overlap of PCOS diagnostic features with normal pubertal physiology. Oligomenorrhoea should not be assessed in the first year following menarche, as this can be a normal part of pubertal development. The criterion for oligomenorrhoea is only met in adolescents if cycles are < 21 or > 45 days for adolescents less than 1 to greater than 3 years post menarche or < 21 or > 35 days or less than 8 cycles per year in those who are more than 3 years post menarche. PCOM can be seen in up to 40% of healthy adolescents at 2 years post menarche (67), and therefore PCOM should not be used as a criterion for PCOS diagnosis within 8 years of menarche (24) to reduce the risk of overdiagnosing PCOS in adolescents. AMH levels are not recommended for diagnosis of PCOS in adolescents as AMH levels only peak in a woman's mid-20s (16). Acne is commonly seen in adolescence and mild acne should not be considered to be a sign of clinical hyperandrogenism for PCOS diagnosis in adolescents. Therefore, diagnosis of PCOS in adolescents requires menstrual cycle irregularity, well-defined according to time post menarche, and either clinical hyperandrogenism (hirsutism and/or severe acne) or biochemical hyperandrogenism as the 2 essential criteria that need to be met (24). Adolescents meeting 1 of the 2 adolescent PCOS diagnostic criterion may be considered “at risk” of PCOS and benefit from follow-up and reassessment 8 years post menarche, in addition to symptom management (11).

## Following Diagnosis

Insulin resistance occurs in approximately 50% to 80% of women with PCOS (68), with intrinsic insulin resistance seen in up to 75% of lean women with PCOS on euglycemic hyperinsulinemic clamp studies, compounded by an extrinsic insulin resistance seen in obese women with PCOS (69). While insulin resistance is a key feature of PCOS, it is not required for PCOS diagnosis, in part due to lack of accurate methods to measure insulin resistance in clinical practice. Women with PCOS are at a 3.3-fold and 2.9-fold increased risk of developing impaired glucose tolerance and type 2 diabetes respectively (70). Women with PCOS should have metabolic screening at diagnosis, including a lipid profile and glycemic testing. Screening tests for dysglycemia include 75-g oral glucose tolerance test (OGTT), fasting glucose, or HbA1c. An OGTT is the recommended method of testing (24), acknowledging cost, access, and inconvenience, as fasting plasma glucose is less accurate in predicting impaired glucose tolerance and type 2 diabetes in these women (71, 72) and HbA1c is a relatively poor diagnostic marker of type 2 diabetes in women with PCOS (72, 73).

## Clinical Cases

### Clinical Case 1

Patient 1, a 17-year-old high school student, was referred by her general practitioner due to primary amenorrhea. She had breast development at the age of 12, pubic and axillary hair at age 12, and experienced a growth spurt at the age of 13. Her older sister and mother had menarche aged 14. She had never taken any hormonal medication and had never been sexually active. There were some significant stressors over the last 2 years with her family situation and she was currently in the final year of high school with significant associated pressure and stress. She had no acne, had mild hirsutism, which was probably in keeping with her Sri Lankan heritage. Her weight was 85.6 kg, with a height of 166 cm, giving a BMI of 31.1 kg/m^2^. Her breast and pubic hair were both Tanner stage 4. They were no stigmata suggestive of Cushing syndrome. Total testosterone, measured using liquid chromatography–mass spectrometry was 1.1 nmol/L (reference range, < 1.8 nmol/L), with a SHBG level of 11 nmol/L (reference range, 25-150 nmol/L) and calculated free testosterone of 33 pmol/L (reference range, 1-34 pmol/L). In excluding secondary causes, her prolactin, thyroid function, and 17-hydroxyprogesterone (17-OHP) levels were normal. Her karyotype was 46 X,X. The FSH level was 4.9 IU/L (reference range, 2-10 IU/L), LH was 2.0 IU/L (reference range, 2-10 IU/L), estradiol (by liquid chromatography–mass spectrometry) 59 pmol/L (reference range, 10-900 pmol/L), and the remaining pituitary panel was unremarkable. Magnetic resonance imaging of the pituitary revealed a normal pituitary gland. Given the primary amenorrhea, a transabdominal pelvic ultrasound was performed, revealing a small uterus measuring 44 × 13 × 23 mm (the average dimensions of the uterus in an nulliparous adult female are 6-8.5 cm long, 3-5 cm wide, and 2-4 cm deep (74)), with a uterine body to cervix ratio approximately 1.5:1, normal myometrium, and thin endometrium with combined thickness of 1.2 mm (2-16 mm depending of stage of menstrual cycle in a premenopausal female (74, 75)). The ovaries were small, with the right ovary measuring 24 mm × 13 mm × 18 mm (volume 3.0 cc) and containing at least 5 small follicles and the left ovary measuring 18 mm × 14 mm × 17 mm (volume 2.2 cc) and containing 5 small follicles. A provisional diagnosis of functional hypothalamic amenorrhea was made and Estradot 25 mcg patch was commenced with the plan to increase the estrogen dose and add in progesterone after the first breakthrough bleed. If the patient's secondary sexual characteristics were not as well developed, a lower dose of transdermal estrogen would have been commenced. The patient currently does not require contraception. The differential diagnosis here is polycystic ovary syndrome. In adolescents, PCOM is not recommended for PCOS diagnosis within 8 years of menarche given the overlap with normal developmental physiology. A diagnosis of PCOS was unable to be made at this stage on the basis of primary amenorrhea alone, in the absence of biochemical hyperandrogenism and convincing clinical hyperandrogenism. This patient should be considered at an “increased risk” of PCOS and reassessment should be considered at full reproductive maturity, 8 years post menarche. The management of adolescents at risk of PCOS is similar to adult women with a clear PCOS diagnosis. For this patient, her HbA1c was 5.9% and she was commenced on metformin and referred to a dietitian for lifestyle advice. In addition, she began a course of intensive psychotherapy with a psychologist.

### Clinical Case 2

Patient 2 is a 21-year-old woman presenting with oligomenorrhea. Menarche occurred at age 13, and she commenced oral contraceptive (OC) therapy 2 years later due to irregular menstrual cycles. She discontinued OCs one year later. The patient has low self-esteem and experiences depressive episodes. She weighs 134 kilograms with a BMI of 42 kg/m^2^ (class III obesity) according to the National Institute of Health and World Health Organization. There are no clinical signs of hirsutism, with a mFG score of 2 (reference range, < 4-6). She had a normal blood pressure of 124/80 mmHg. Fasting laboratory results indicated a FSH level of 7.0 IU/L (reference range, 2.0-10.0 IU/L), LH level of 8.7 IU/L (reference range, 2.0-10.0 IU/L), and an estradiol level of 160 pmol/L (reference range, 100-900 pmol/L). Thyroid function test and androgen levels were within normal ranges. She had a fasting glucose level of 5.5 mmol/L (reference range, 4.0-5.6 mmol/L). Ultrasound of the ovaries was inconclusive due to severe obesity. Following the diagnostic algorithm as presented by the 2023 International Guideline, an AMH level was measured and was found to be elevated at 52.8 pmol/L (reference ranges are based on population- and assay-specific cutoffs (64)). The diagnosis of PCOS was made based on the presence oligomenorrhea and elevated AMH levels. A 75-g OGTT yielded normal results. The patient underwent a gastric bypass and subsequently lost over 30 kilograms within the following year.

### Clinical Case 3

Patient 3, a 27-year-old woman, was referred by her dermatologist due to excessive hair growth. Menarche occurred at 11 years of age, following which her general practitioner prescribed an OC to manage the excessive blood loss during her periods. Three years ago, she discontinued OCs due to mood swings. Her menstrual cycle is regular with a cycle length of 27 days, ranging from 26 to 30 days. After discontinuation of OCs, her symptoms of hirsutism, alopecia, and acne exacerbated. Her BMI was 26.6 kg/m^2^ and she experienced a weight gain of 5 kilograms in the past year. Her mFG score was 10 (reference range, < 4-6). Results of the transvaginal ultrasound of the third cycle day described an anteverted uterus of normal size, the right ovary was 8.8 mL (reference range, < 10 mL) with 28 antral follicles (reference range for PCOM > 20 pre-ovulatory or antral follicles), the left ovary was 6.1 mL with 40 antral follicles. No masses were observed, no free fluid was present. Laboratory investigations revealed FSH level of 6.3 IU/L (reference range, 2.0-10.0 IU/L), LH level of 8.8 IU/L (reference range, 2.0-10.0 IU/L), estradiol of 153 pmol/L (reference range, 100-900 pmol/L), and normal cortisol levels. Her FAI was 5.4 (reference range, 0.2-5.4). She had a fasting glucose level of 5.1 mmol/L (reference range, 4.0-5.6 mmol/L). Based on clinical and biochemical hirsutism and PCOM on ultrasound, a diagnosis of PCOS was established. She was advised to resume OCs and laser hair removal for management of hirsutism; however, she was reluctant to recommence an OC due to previous mood adverse effects. All available OC pills have the potential of a deleterious effect on mood. A small pilot study has shown that OCs containing estradiol and nomegestrol may be better tolerated by women with mood disorders (76). An option for this patient would be to trial an OC containing estradiol and nomegestrol. Addition of an anti-androgen could be considered, ideally following 6 months of OC therapy and depending on response to laser hair removal.

### Clinical Case 4

Patient 4 is a 23-year-old woman who complains of unwanted hair growth on her face and body and increased shedding and thinning of her scalp hair. She reports regular “periods” on a more or less monthly basis. History indicates that the excess hair growth began when the patient was in high school and has been getting progressively worse, especially after discontinuing OCs, which had been prescribed for irregular cycles, some 18 months earlier. She is not currently sexually active. Her scalp hair loss began shortly after she discontinued OCs. Social and family history are unremarkable. Physical examination reveals a normal weight woman in no acute distress, with normal blood pressure, normal thyroid on palpation, no evidence of Cushingoid features, no galactorrhea, and an mFG score of 6, with terminal hair growth mostly on the chin, neck, and lower abdomen. Examination of the scalp reveals few hairs (1-3 per pull) in telogen during the hair pull test (23), although she had brushed her hair earlier in the day, and no obvious areas of hair loss or baldness.

Evaluation included assessing 17-OHP obtained in the morning in the pre-ovulatory or follicular phase of the menstrual cycle (ie, days 3-8) following a spontaneous vaginal bleed or period, and for convenience, measuring TSH and prolactin at the same time. The patient also underwent the measurement of a progesterone (P4) level on days 22-24 of the menstrual cycle and a transvaginal ultrasound to assess the ovarian morphology. TSH and prolactin were normal, and 17-OHP was < 6.06 nmol/L. Transvaginal ultrasound indicated PCOM and the progesterone level at days 22 to 24 was < 9.54 nmol/L. In this case, we have a patient with hirsutism of a moderate degree, PCOM, and evidence of anovulation, and no evidence of thyroid dysfunction, hyperprolactinemia, and 21-hydroxylase deficient NCAH.

The maximum cutoff value for a baseline 17-OHP level to exclude 21-OH deficient NCAH is 6 nmol/L (77). We should note that that the baseline 17-OHP to exclude NCAH should be obtained in the follicular phase, within a few days of starting a spontaneous or progestin-induced vaginal bleed and preferably in the morning. The levels of 17-OHP decrease throughout the day and can be spuriously elevated in the luteal (postovulatory) phase (77). Furthermore, while a history or regular vaginal bleeding in non-hirsute women is highly predictive of regular ovulation, especially if accompanied by premenstrual molimina symptoms (eg, mood changes, generalized swelling, or breast tenderness), about 40% of patients who claim to have regular “periods” actually have regularly occurring breakthrough bleeding, but are anovulatory (78).

The patient restarted OCs and, as OCs alone had not been sufficient to control her unwanted hair in the past, an anti-androgen, spironolactone 100 mg/day, was added to the treatment regimen. In follow-up, the patient noted she was experiencing regular vaginal bleeding and decreasing facial and chest hair growth, stable scalp hair shedding, and was satisfied with her care.

### Clinical Case 5

Patient 5 is a 54-year-old woman, presenting with worsening hirsutism. Her medical history included hypertension, dyslipidemia, and peripheral vascular disease, with her only surgical intervention being an ovary-sparing hysterectomy for menorrhagia approximately a decade ago. She was on a regimen of daily ramipril 5 mg, rosuvastatin 5 mg, and aspirin 100 mg. She reported regular menstrual cycles until her hysterectomy, after which she experienced an escalation in her hirsutism and noticed an increased frequency of shaving over the past 18 months. Additionally, she mentioned experiencing intermittent vasoactive hot flushes over the last 2 years.

Upon examination, the patient presented with significant virilization, evidenced by excessive hair growth on her face, arms, and legs, and a mFG score of 19. Despite being overweight with a BMI of 28.7 kg/m^2^, no features of Cushingoid appearance or abdominal striae were observed, and clitoromegaly was absent.

Further investigations were pursued to elucidate the source of hyperandrogenism. Computed tomography adrenal imaging incidentally discovered a 13-mm lipid-rich adenoma for which subsequent evaluation of aldosterone to renin ratio (after replacing ramipril to verapamil), plasma metanephrines, and a 1-mg dexamethasone suppression test confirmed the nonfunctioning nature of the adrenal lesion. No ovarian tumors were detected on pelvic ultrasound and computed tomography. It is important to note that many androgen-=secreting tumors are often small and difficult to detect on imaging of the ovaries. Androgen profiling revealed persistent hyperandrogenemia with elevated total testosterone levels (5.5 nmol/L) (reference range, 0.1-1.7 nmol/L), an elevated FAI of 24.9 (reference range, 0.2-5.4) and elevated androstenedione levels of 5.2 nmol/L (reference range, 0.5-2.9 nmol/L). DHEAS remained within normal postmenopausal limits at 1.0 umol/L (reference range, 0.2-5.1 umol/L). Additionally, the patient exhibited elevated LH and FSH levels consistent with the postmenopausal range. Secondary causes of hyperandrogenemia were excluded with a 17-OHP level of 3.2 nmol/L (reference range, <6.0 nmol/L) and normal thyroid function tests.

Overall, the patient's marked hyperandrogenemia, postmenopausal status, history of regular menstrual cycles and absence of PCOM, indicates an alternative diagnosis rather than PCOS. To investigate the source of the excess testosterone, combined adrenal and ovarian vein sampling was undertaken which confirmed bilateral ovarian hypersecretion of testosterone. Given the bilateral hypersecretion, a diagnosis of ovarian hyperthecosis is most likely diagnosis (as opposed to an androgen-secreting ovarian tumor, which is more likely to be unilateral). Consequently, the patient was referred to a gynecologist for consideration of bilateral oophorectomy.

### Clinical Case 6

Patient 6 is a 23-year-old dancing instructor, who was referred for investigation of secondary amenorrhea. She had a previous ankle injury but was otherwise healthy. She reported a history of irregular periods since menarche at age 15, often occurring every couple of months. Acne during her teenaged years was managed with topical therapy. The patient followed a mainly pescatarian diet and maintained an active lifestyle, working full-time as a dancing instructor, walking for 1 hour daily, and engaging in resistance training for 1 hour 5 days a week. On examination, she appeared lean with a BMI of 18.7 kg/m^2^. While she had apparent vellus hair, hirsutism was not present. Clinical suspicion for hypothalamic hypogonadism was high and indeed her pituitary hormone panel revealed undetectable FSH, LH, and estradiol. Further investigations to exclude PCOS included normal ovarian ultrasound, total testosterone levels, and FAI.

The patient was counseled on her diagnosis and advised to reduce energy expenditure and gain weight. She was referred to a dietitian for personalized nutritional counseling to ensure a balanced diet for weight gain while maintaining overall health. Additionally, she underwent bone health assessment, revealing normal bone mineral density but elevated bone turnover markers, indicating heightened bone resorption. After discussing treatment options, the patient was started on hormone replacement therapy to promote bone health. Over the course of 3 years, the patient adhered to the treatment plan and as she successfully gained weight, her hormonal balance was restored, and she regained normal menstrual cycles without hormone replacement therapy.

## Conclusions

PCOS is a complex condition and one of the most frequent endocrine disorders, affecting 10% to 13% of all women. The disorder has significant economic burden (79-81) and cardiometabolic, reproductive, mental health, and quality of life consequences (82) and, while lifelong, is most clinically evident in reproductive-aged women. There is substantial need for improved awareness and knowledge among clinicians regarding this highly prevalent and morbid disorder, with high degrees of patient dissatisfaction concerning timely diagnosis and care.

There are a number of remaining challenges and uncertainties to the diagnosis of PCOS. PCOS continues to be under-recognized by patients and clinicians alike (83-86), which can only be addressed by a significant level of educational resources and commitment. The global availability (or lack of availability) of high-quality ovarian ultrasonography, as well as androgen and AMH assays, remains a significant obstacle to the evaluation of patients with suspected PCOS worldwide. More research needs to be done determining the definition and diagnosis of hirsutism in various ethnic populations, to develop more readily available assays for androgen and AMH measurements and to understand the predictive value and diagnostic cutoffs for AMH in various age groups and ethnicities in detecting PCOM.

## Data Availability

Data sharing is not applicable to this article as no datasets were generated or analyzed during the current study.

## Abbreviations

17-OHP: 17-hydroxyprogesterone
AMH: anti-Müllerian hormone
BMI: body mass index
DHEAS: dehydroepiandrosterone sulfate
FAI: free androgen index
FNPO: follicle number per ovary
FSH: follicle stimulating hormone
LH: luteinizing hormone
NCAH: non-classic congenital adrenal hyperplasia
OC: oral contraceptive
OGTT: oral glucose tolerance test
PCOM: polycystic ovary morphology
PCOS: polycystic ovary syndrome
SHBG: sex hormone binding globulin
TSH: thyrotropin (thyroid stimulating hormone)

## References

[1] Bozdag G, Mumusoglu S, Zengin D, Karabulut E, Yildiz BO. The prevalence and phenotypic features of polycystic ovary syndrome: a systematic review and meta-analysis. Hum Reprod. 2016;31(12):2841‐2855. 27664216 10.1093/humrep/dew218

[2] Naz MSG, Tehrani FR, Majd HA, et al The prevalence of polycystic ovary syndrome in adolescents: a systematic review and meta-analysis. Int J Reprod Biomed. 2019;17:533‐542. 31583370 10.18502/ijrm.v17i8.4818 PMC6745085

[3] Kakoly NS, Earnest A, Teede HJ, Moran LJ, Joham AE. The impact of obesity on the incidence of type 2 diabetes among women with polycystic ovary syndrome. Diabetes Care. 2019;42(4):560‐567. 30705063 10.2337/dc18-1738

[4] Joham A, Teede HJ, Ranasinha S, Zoungas S, Boyle J. Infertility and assisted reproductive technology use in women with polycystic ovary syndrome: data from the Australian Longitudinal Women's Health Study. J Women's Health. 2015;24(4):299‐307. 10.1089/jwh.2014.5000 25654626

[5] Carmina E, Azziz R, Bergfeld W, et al Female pattern hair loss and androgen excess: a report from the multidisciplinary androgen excess and PCOS committee. J Clin Endocrinol Metab. 2019;104(7):2875‐2891. 30785992 10.1210/jc.2018-02548

[6] Cooney LG, Lee I, Sammel MD, Dokras A. High prevalence of moderate and severe depressive and anxiety symptoms in polycystic ovary syndrome: a systematic review and meta-analysis. Hum Reprod. 2017;32(5):1075‐1091. 28333286 10.1093/humrep/dex044

[7] Tay CT, Teede HJ, Hill B, Loxton D, Joham AE. Increased prevalence of eating disorders, low self-esteem, and psychological distress in women with polycystic ovary syndrome: a community-based cohort study. Fertil Steril. 2019;112(2):353‐361. 31056307 10.1016/j.fertnstert.2019.03.027

[8] Tay CT, Teede HJ, Loxton D, Kulkarni J, Joham AE. Psychiatric comorbidities and adverse childhood experiences in women with self-reported polycystic ovary syndrome: an Australian population-based study. Psychoneuroendocrinology. 2020;116:104678. 32361187 10.1016/j.psyneuen.2020.104678

[9] Karjula S, Morin-Papunen L, Franks S, et al Population-based data at ages 31 and 46 show decreased HRQoL and life satisfaction in women with PCOS symptoms. J Clin Endocrinol Metab. 2020;105(6):1814‐1826. 31970392 10.1210/clinem/dgz256 PMC7150615

[10] Rotterdam ESHRE/ASRM-Sponsored PCOS consensus workshop group . Revised 2003 consensus on diagnostic criteria and long-term health risks related to polycystic ovary syndrome (PCOS). Hum Reprod 2004; 19(1):41‐47 14688154 10.1093/humrep/deh098

[11] Teede HJ, Tay CT, Laven J, et al Evidence-based Guideline for the Assessment and Management of Polycystic Ovary Syndrome. Monash University; 2023.

[12] Pall M, Azziz R, Beires J, Pignatelli D. The phenotype of hirsute women: a comparison of polycystic ovary syndrome and 21-hydroxylase-deficient nonclassic adrenal hyperplasia. Fertil Steril. 2010;94(2):684‐689. 19726039 10.1016/j.fertnstert.2009.06.025

[13] Azziz R, Carmina E, Dewailly D, et al Position statement: criteria for defining polycystic ovary syndrome as a predominantly hyperandrogenic syndrome: an androgen excess society guideline. J Clin Endocrinol Metab. 2006;91(11):4237‐4245. 16940456 10.1210/jc.2006-0178

[14] Lizneva D, Suturina L, Walker W, Brakta S, Gavrilova-Jordan L, Azziz R. Criteria, prevalence, and phenotypes of polycystic ovary syndrome. Fertil Steril. 2016;106(1):6‐15. 27233760 10.1016/j.fertnstert.2016.05.003

[15] Azziz R, Waggoner WT, Ochoa T, Knochenhauer ES, Boots LR. Idiopathic hirsutism: an uncommon cause of hirsutism in Alabama. Fertil Steril. 1998;70(2):274‐278. 9696220 10.1016/s0015-0282(98)00141-1

[16] Dewailly D, Andersen CY, Balen A, et al The physiology and clinical utility of anti-Mullerian hormone in women. Hum Reprod Update. 2014;20(3):370‐385. 24430863 10.1093/humupd/dmt062

[17] Stener-Victorin E, Holm G, Labrie F, Nilsson L, Janson PO, Ohlsson C. Are there any sensitive and specific sex steroid markers for polycystic ovary syndrome? J Clin Endocrinol Metab. 2010;95(2):810‐819. 20016048 10.1210/jc.2009-1908

[18] Burgers JA, Fong SL, Louwers YV, et al Oligoovulatory and anovulatory cycles in women with polycystic ovary syndrome (PCOS): what's the difference? J Clin Endocrinol Metab. 2010;95(12):E485‐E489. 20843954 10.1210/jc.2009-2717

[19] Lizneva D, Kirubakaran R, Mykhalchenko K, et al Phenotypes and body mass in women with polycystic ovary syndrome identified in referral versus unselected populations: systematic review and meta-analysis. Fertil Steril. 2016;106(6):1510‐1520.e1512. 27530062 10.1016/j.fertnstert.2016.07.1121

[20] Shroff R, Syrop CH, Davis W, Van Voorhis BJ, Dokras A. Risk of metabolic complications in the new PCOS phenotypes based on the Rotterdam criteria. Fertil Steril. 2007;88(5):1389‐1395. 17462641 10.1016/j.fertnstert.2007.01.032

[21] Goverde AJ, van Koert AJB, Eijkemans MJ, et al Indicators for metabolic disturbances in anovulatory women with polycystic ovary syndrome diagnosed according to the Rotterdam consensus criteria. Hum Reprod. 2009;24(3):710‐717. 19095675 10.1093/humrep/den433

[22] Lerchbaum E, Schwetz V, Giuliani A, Pieber TR, Obermayer-Pietsch B. Opposing effects of dehydroepiandrosterone sulfate and free testosterone on metabolic phenotype in women with polycystic ovary syndrome. Fertil Steril. 2012;98(5):1318‐1325.e1311. 22835450 10.1016/j.fertnstert.2012.07.1057

[23] Lizneva D, Gavrilova-Jordan L, Walker W, Azziz R. Androgen excess: investigations and management. Best Pract Res Clin Obstet Gynaecol. 2016;37:98‐118. 27387253 10.1016/j.bpobgyn.2016.05.003

[24] Teede HJ, Tay CT, Laven J, et al Recommendations from the 2023 international evidence-based guideline for the assessment and management of polycystic ovary syndrome. Fertil Steril. 2023;120(4):767‐793. 37589624 10.1016/j.fertnstert.2023.07.025

[25] Yildiz BO, Bolour S, Woods K, Moore A, Azziz R. Visually scoring hirsutism. Hum Reprod Update. 2010;16(1):51‐64. 19567450 10.1093/humupd/dmp024 PMC2792145

[26] Ferriman D, Gallwey JD. Clinical assessment of body hair growth in women. J Clin Endocrinol Metab. 1961;21(11):1440‐1447. 13892577 10.1210/jcem-21-11-1440

[27] Oliveira TF, Oliveira TF, Reis FM, et al Revisiting the utility of simplified Ferriman-Gallwey score (sFG) to predict hirsutism in Latin American women. Arch Dermatol Res. 2024;316(7):475. 39023750 10.1007/s00403-024-03206-7

[28] Kahraman FC, Erdoğan SS. Grading of hirsutism: a practical approach to the modified Ferriman-Gallwey scoring system. Postepy Dermatol Alergol. 2022;39(4):744‐748. 36090715 10.5114/ada.2021.108455 PMC9454347

[29] Yang Y, Ouyang N, Ye Y, et al The predictive value of total testosterone alone for clinical hyperandrogenism in polycystic ovary syndrome. Reprod Biomed Online. 2020;41(4):734‐742. 32912651 10.1016/j.rbmo.2020.07.013

[30] Yang Y, Han Y, Wang W, et al Assessing new terminal body and facial hair growth during pregnancy: toward developing a simplified visual scoring system for hirsutism. Fertil Steril. 2016;105(2):494‐500. 26616440 10.1016/j.fertnstert.2015.10.036

[31] Ramezani Tehrani F, Minooee S, Azizi F. Validation of a simplified method to assess hirsutism in the Iranian population. Eur J Obstet Gynecol Reprod Biol. 2014;174:91‐95. 24393448 10.1016/j.ejogrb.2013.12.008

[32] Cook H, Brennan K, Azziz R. Reanalyzing the modified Ferriman-Gallwey score: is there a simpler method for assessing the extent of hirsutism? Fertil Steril. 2011;96(5):1266‐1270.e1261. 21924716 10.1016/j.fertnstert.2011.08.022 PMC3205229

[33] Wild RA, Vesely S, Beebe L, Whitsett T, Owen W. Ferriman Gallwey self-scoring I: performance assessment in women with polycystic ovary syndrome. J Clin Endocrinol Metab. 2005;90(7):4112‐4114. 15827102 10.1210/jc.2004-2243

[34] Espinós JJ, Calaf J, Estadella J, Checa MA. Hirsutism scoring in polycystic ovary syndrome: concordance between clinicians' and patients' self-scoring. Fertil Steril. 2010;94(7):2815‐2816. 20579644 10.1016/j.fertnstert.2010.05.022

[35] Pasch L, He SY, Huddleston H, et al Clinician vs self-ratings of hirsutism in patients with polycystic ovarian syndrome: associations with quality of life and depression. JAMA Dermatol. 2016;152(7):783‐788. 26942548 10.1001/jamadermatol.2016.0358

[36] Taponen S, Ahonkallio S, Martikainen H, et al Prevalence of polycystic ovaries in women with self-reported symptoms of oligomenorrhoea and/or hirsutism: Northern Finland Birth Cohort 1966 Study. Hum Reprod. 2004;19(5):1083‐1088. 15044401 10.1093/humrep/deh214

[37] Chan JL, Pall M, Ezeh U, Mathur R, Pisarska MD, Azziz R. Screening for androgen excess in women: accuracy of self-reported excess body hair growth and menstrual dysfunction. J Clin Endocrinol Metab. 2020;105(10):e3688‐e3695. 32442282 10.1210/clinem/dgz264 PMC7448931

[38] Gabrielli L, Aquino EML. A simplified questionnaire for self-assessment of hirsutism in population-based studies. Eur J Endocrinol. 2015;172(4):451‐459. 25583904 10.1530/EJE-14-0591

[39] Martin KA, Anderson RR, Chang RJ, et al Evaluation and treatment of hirsutism in premenopausal women: an Endocrine Society clinical practice guideline. J Clin Endocrinol Metab. 2018;103(4):1233‐1257. 29522147 10.1210/jc.2018-00241

[40] Ferriman D, Purdie AW. The aetiology of oligomenorrhoea and/or hirsuties: a study of 467 patients. Postgrad Med J. 1983;59(687):17‐20. 6866869 10.1136/pgmj.59.687.17 PMC2417350

[41] DeUgarte CM, Woods KS, Bartolucci AA, Azziz R. Degree of facial and body terminal hair growth in unselected black and white women: toward a populational definition of hirsutism. J Clin Endocrinol Metab. 2006;91(4):1345‐1350. 16449347 10.1210/jc.2004-2301

[42] Zhao X, Ni R, Li L, et al Defining hirsutism in Chinese women: a cross-sectional study. Fertil Steril. 2011;96(3):792‐796. 21762890 10.1016/j.fertnstert.2011.06.040

[43] Kim JJ . Update on polycystic ovary syndrome. Clin Exp Reprod Med. 2021;48(3):194‐197. 34488284 10.5653/cerm.2020.04329 PMC8421664

[44] Kiconco S, Mousa A, Azziz R, et al PCOS Phenotype in Unselected Populations Study (P-PUP): protocol for a systematic review and defining PCOS diagnostic features with pooled individual participant data. Diagnostics (Basel). 2021;11(11):1953 34829300 10.3390/diagnostics11111953 PMC8618006

[45] Pace L, Kummer N, Wallace M, Azziz R. The value of androgen measures for diagnosing polycystic ovarian syndrome (PCOS) in an unselected population. Reprod Sci. 2025;32(1):168‐175. 39419927 10.1007/s43032-024-01702-9 PMC11729065

[46] Grassi G, Polledri E, Fustinoni S, et al Hyperandrogenism by liquid chromatography tandem mass spectrometry in PCOS: focus on testosterone and androstenedione. J Clin Med. 2020:10:119. 33396396 10.3390/jcm10010119 PMC7795755

[47] Miller KK, Rosner W, Lee H, et al Measurement of free testosterone in normal women and women with androgen deficiency: comparison of methods. J Clin Endocrinol Metab. 2004;89(2):525‐533. 14764757 10.1210/jc.2003-030680

[48] Sathyapalan T, Al-Qaissi A, Kilpatrick ES, et al Salivary testosterone measurement in women with and without polycystic ovary syndrome. Sci Rep. 2017;7(1):3589. 28620242 10.1038/s41598-017-03945-w PMC5472559

[49] Keevil BG, Adaway J, Fiers T, Moghetti P, Kaufman J-M. The free androgen index is inaccurate in women when the SHBG concentration is low. Clin Endocrinol (Oxf). 2018;88(5):706‐710. 29405348 10.1111/cen.13561

[50] Vermeulen A, Verdonck L, Kaufman JM. A critical evaluation of simple methods for the estimation of free testosterone in serum. J Clin Endocrinol Metab. 1999;84(10):3666‐3672. 10523012 10.1210/jcem.84.10.6079

[51] Rosner W, Auchus RJ, Azziz R, Sluss PM, Raff H. Position statement: utility, limitations, and pitfalls in measuring testosterone: an Endocrine Society position statement. J Clin Endocrinol Metab. 2007;92(2):405‐413. 17090633 10.1210/jc.2006-1864

[52] Goldman AL, Bhasin S, Wu FCW, Krishna M, Matsumoto AM, Jasuja R. A reappraisal of Testosterone's binding in circulation: physiological and clinical implications. Endocr Rev. 2017;38(4):302‐324. 28673039 10.1210/er.2017-00025 PMC6287254

[53] Handelsman DJ . Free testosterone: pumping up the tires or ending the free ride? Endocr Rev. 2017;38(4):297‐301. 28898980 10.1210/er.2017-00171

[54] Huang A, Brennan K, Azziz R. Prevalence of hyperandrogenemia in the polycystic ovary syndrome diagnosed by the National Institutes of Health 1990 criteria. Fertil Steril. 2010;93(6):1938‐1941. 19249030 10.1016/j.fertnstert.2008.12.138 PMC2859983

[55] O’Reilly MW, Kempegowda P, Jenkinson C, et al 11-Oxygenated C19 steroids are the predominant androgens in polycystic ovary syndrome. J Clin Endocrinol Metab. 2017;102(3):840‐848. 27901631 10.1210/jc.2016-3285 PMC5460696

[56] Torchen LC, Sisk R, Legro RS, Turcu AF, Auchus RJ, Dunaif A. 11-Oxygenated C19 steroids do not distinguish the hyperandrogenic phenotype of PCOS daughters from girls with obesity. J Clin Endocrinol Metab. 2020;105(11):e3903‐e3909. 32797203 10.1210/clinem/dgaa532 PMC7500474

[57] Tosi F, Villani M, Garofalo S, et al Clinical value of Serum levels of 11-oxygenated metabolites of testosterone in women with polycystic ovary syndrome. J Clin Endocrinol Metab. 2022;107(5):e2047‐e2055. 34951635 10.1210/clinem/dgab920

[58] Dewailly D, Lujan ME, Carmina E, et al Definition and significance of polycystic ovarian morphology: a task force report from the Androgen Excess and Polycystic Ovary Syndrome Society. Hum Reprod Update. 2014;20(3):334‐352. 24345633 10.1093/humupd/dmt061

[59] Rosenfield RL . The polycystic ovary morphology-polycystic ovary syndrome spectrum. J Pediatr Adolesc Gynecol. 2015;28(6):412‐419. 25840648 10.1016/j.jpag.2014.07.016 PMC4387116

[60] Pea J, Bryan J, Wan C, et al Ultrasonographic criteria in the diagnosis of polycystic ovary syndrome: a systematic review and diagnostic meta-analysis. Hum Reprod Update. 2024;30(1):109‐130. 37804097 10.1093/humupd/dmad027 PMC10762001

[61] Lazareva L, Suturina L, Atalyan A, et al Ovarian morphology in non-hirsute, normo-androgenic, eumenorrheic premenopausal women from a multi-ethnic unselected Siberian population. Diagnostics (Basel). 2024;14(7):673. 38611586 10.3390/diagnostics14070673 PMC11012196

[62] Teede H, Misso M, Tassone EC, et al Anti-Müllerian hormone in PCOS: a review informing international guidelines. Trends Endocrinol Metab. 2019;30(7):467‐478. 31160167 10.1016/j.tem.2019.04.006

[63] Fraissinet A, Robin G, Pigny P, Lefebvre T, Catteau-Jonard S, Dewailly D. Use of the serum anti-Müllerian hormone assay as a surrogate for polycystic ovarian morphology: impact on diagnosis and phenotypic classification of polycystic ovary syndrome. Hum Reprod. 2017;32(8):1716‐1722. 28854589 10.1093/humrep/dex239

[64] van der Ham K, Laven JSE, Tay CT, Mousa A, Teede H, Louwers YV. Anti-Müllerian hormone as a diagnostic biomarker for polycystic ovary syndrome and polycystic ovarian morphology: a systematic review and meta-analysis. Fertil Steril. 2024;122(4):727‐739. 38944177 10.1016/j.fertnstert.2024.05.163

[65] van der Ham K, Barbagallo F, van Schilfgaarde E, Lujan ME, Laven JSE, Louwers YV. The additional value of ultrasound markers in the diagnosis of polycystic ovary syndrome. Fertil Steril. Published online August 31, 2024. Doi: 10.1016/j.fertnstert.2024.08.342 39218282

[66] Barbagallo F, van der Ham K, Willemsen SP, Louwers YV, Laven JS. Age-related curves of AMH using the Gen II, the picoAMH, and the elecsys assays in women with polycystic ovary syndrome. J Clin Endocrinol Metab. 2024;109(10):2561‐2570. 38486510 10.1210/clinem/dgae153 PMC11403310

[67] Meczekalski B, Niwczyk O, Kostrzak A, Maciejewska-Jeske M, Bala G, Szeliga A. PCOS in adolescents-ongoing riddles in diagnosis and treatment. J Clin Med. 2023;12(3):1221. 36769869 10.3390/jcm12031221 PMC9918268

[68] Teede H, Deeks A, Moran L. Polycystic ovary syndrome: a complex condition with psychological, reproductive and metabolic manifestations that impacts on health across the lifespan. BMC Med. 2010;8(1):41. 20591140 10.1186/1741-7015-8-41 PMC2909929

[69] Stepto NK, Cassar S, Joham AE, et al Women with polycystic ovary syndrome have intrinsic insulin resistance on euglycaemic-hyperinsulaemic clamp. Hum Reprodr. 2013;28(3):777‐784. 10.1093/humrep/des463 23315061

[70] Kakoly NS, Khomami MB, Joham AE, et al Ethnicity, obesity and the prevalence of impaired glucose tolerance and type 2 diabetes in PCOS: a systematic review and meta-regression. Hum Reprod Update. 2018;24(4):455‐467. 29590375 10.1093/humupd/dmy007

[71] Ortiz-Flores AE, Luque-Ramírez M, Fernández-Durán E, Alvarez-Blasco F, Escobar-Morreale HF. Diagnosis of disorders of glucose tolerance in women with polycystic ovary syndrome (PCOS) at a tertiary care center: fasting plasma glucose or oral glucose tolerance test? Metabolism. 2019;93:86‐92. 30710572 10.1016/j.metabol.2019.01.015

[72] Lerchbaum E, Schwetz V, Giuliani A, Obermayer-Pietsch B. Assessment of glucose metabolism in polycystic ovary syndrome: Hba1c or fasting glucose compared with the oral glucose tolerance test as a screening method. Hum Reprod. 2013;28(9):2537‐2544. 23756702 10.1093/humrep/det255

[73] Velling Magnussen L, Mumm H, Andersen M, Glintborg D. Hemoglobin A1c as a tool for the diagnosis of type 2 diabetes in 208 premenopausal women with polycystic ovary syndrome. Fertil Steril. 2011;96(5):1275‐1280. 21982282 10.1016/j.fertnstert.2011.08.035

[74] Chaoui R, Jeanty P, Paladini D. Ultrasound of the non-pregnant uterus. In: Abuhamad A, ed. Ultrasound in Obstetrics and Gynecology: A Practical Approach. 1st ed. 2014:217‐223.

[75] Tsuda H, Ito YM, Todo Y, et al Measurement of endometrial thickness in premenopausal women in office gynecology. Reprod Med Biol. 2018;17(1):29‐35. 29371818 10.1002/rmb2.12062 PMC5768977

[76] Robertson E, Thew C, Thomas N, Karimi L, Kulkarni J. Pilot data on the feasibility and clinical outcomes of a nomegestrol acetate oral contraceptive pill in women with premenstrual dysphoric disorder. Front Endocrinol (Lausanne). 2021;12:704488. 34630323 10.3389/fendo.2021.704488 PMC8498579

[77] Azziz R, Hincapie LA, Knochenhauer ES, Dewailly D, Fox L, Boots LR. Screening for 21-hydroxylase-deficient nonclassic adrenal hyperplasia among hyperandrogenic women: a prospective study. Fertil Steril. 1999;72(5):915‐925. 10561000 10.1016/s0015-0282(99)00383-0

[78] Azziz R, Carmina E, Sawaya ME. Idiopathic hirsutism. Endocr Rev. 2000;21(4):347‐362. 10950156 10.1210/edrv.21.4.0401

[79] Azziz R, Marin C, Hoq L, Badamgarav E, Song P. Health care-related economic burden of the polycystic ovary syndrome during the reproductive life span. J Clin Endocrinol Metab. 2005;90(8):4650‐4658. 15944216 10.1210/jc.2005-0628

[80] Riestenberg C, Jagasia A, Markovic D, Buyalos RP, Azziz R. Health care-related economic burden of polycystic ovary syndrome in the United States: pregnancy-related and long-term health consequences. J Clin Endocrinol Metab. 2022;107(2):575‐585. 34546364 10.1210/clinem/dgab613

[81] Yadav S, Delau O, Bonner AJ, et al Direct economic burden of mental health disorders associated with polycystic ovary syndrome: systematic review and meta-analysis. Elife. 2023;12:e85338. 37534878 10.7554/eLife.85338 PMC10471160

[82] Zore T, Joshi N, Lizneva D, Azziz R. Polycystic ovarian syndrome: long-term health consequences. Semin Reprod Med. 2017;35(3):271‐281. 28658711 10.1055/s-0037-1603096

[83] Ismayilova M, Yaya S. “I felt like she didn’t take me seriously”: a multi-methods study examining patient satisfaction and experiences with polycystic ovary syndrome (PCOS) in Canada. BMC Womens Health. 2022;22(1):47. 35197027 10.1186/s12905-022-01630-3 PMC8864824

[84] Gibson-Helm M, Dokras A, Karro H, Piltonen T, Teede H. Knowledge and practices regarding polycystic ovary syndrome among physicians in Europe, North America, and internationally: an online questionnaire-based study. Semin Reprod Med. 2018;36(1):19‐27. 30189447 10.1055/s-0038-1667155

[85] Dokras A, Saini S, Gibson-Helm M, Schulkin J, Cooney L, Teede H. Gaps in knowledge among physicians regarding diagnostic criteria and management of polycystic ovary syndrome. Fertil Steril. 2017;107(6):1380‐1386.e1381. 28483503 10.1016/j.fertnstert.2017.04.011

[86] Gibson-Helm M, Teede H, Dunaif A, Dokras A. Delayed diagnosis and a lack of information associated with dissatisfaction in women with polycystic ovary syndrome. J Clin Endocrinol Metab. 2016;102:604‐612. 10.1210/jc.2016-2963 PMC6283441 27906550

